# Metabolic Healthy Obesity, Vitamin D Status, and Risk of COVID-19

**DOI:** 10.14336/AD.2020.1108

**Published:** 2021-02-01

**Authors:** Shu Li, Zhi Cao, Hongxi Yang, Yuan Zhang, Fusheng Xu, Yaogang Wang

**Affiliations:** ^1^School of Public Health, Tianjin Medical University, Tianjin, China.; ^2^School of Public Health, Yale University, New Haven, CT, USA.

**Keywords:** Obesity, metabolic health, vitamin D, COVID-19

## Abstract

Aging and obesity-related conditions seem to worsen the effect of Coronavirus Disease 2019 (COVID-19). This study assessed the possible roles of metabolic/obesity phenotypes and vitamin D status in increasing the greater severity of COVID-19. We studied 353,299 UK Biobank participants from England with a mean age of 67.7 years. Metabolic/obesity phenotypes were defined as a combination of metabolic components (hypertension, high cholesterol, and diabetes) and obesity. Multivariate logistic regression analysis was performed to test whether the addition of metabolic disorders and vitamin D insufficiency increased obesity associations with COVID-19 hospitalization, confirmed COVID-19, and severe COVID-19. Metabolically unhealthy obesity (MUHO) represented 12.3% of the total analytic samples, and 21.5%, 18.5%, and 19.8% of the included subpopulations with COVID-19 hospitalization, confirmed COVID-19, and severe COVID-19, respectively. Vitamin D insufficiency phenotypes represented 53.5% of the total analytic samples, and 59.5%, 61.7%, and 61.5% of the included subpopulations with COVID-19 hospitalization, confirmed COVID-19, and severe COVID-19, respectively. In multivariate logistic regression, MUHO and vitamin D insufficiency and their combination were significantly associated with COVID-19 illness severity (odds ratio [OR] for COVID-19 hospitalization = 2.33, 95% confidence interval [CI], 2.02-2.70; OR for confirmed COVID-19 = 2.06, 95% CI, 1.58-2.70; OR for severe COVID-19 = 2.06, 95% CI, 1.47-2.87). Elderly men were prone to have a higher risk of COVID-19 than women. Our findings showed that MUHO and vitamin D insufficiency are associated with a significantly increased risk of COVID-19 severity, especially for adults 65 years and older. Susceptible individuals should be aware of their conditions and avoid contact with new coronavirus.

Coronavirus Disease 2019 (COVID-19), which is caused by the severe acute respiratory syndrome coronavirus 2 (SARS-CoV-2), is threatening human health worldwide [1]. As of July 31, 2020, approximately 17.45 million cases and 655,112 deaths have been confirmed worldwide (https://www.worldometers.info/coronavirus/ accessed on July 31, 2020). People of any age are susceptible to SARS-CoV-2 and have shown a differential pattern of disease severity. Various preexisting conditions have been proposed as risk factors for the initial infection and subsequent poor health outcomes. Potential explanations for the risk of severe illness from COVID-19 include, but are not limited to, older age, social vulnerability and economic status, severe obesity and having underlying diseases [2].

Studies have reported that adults with a high body mass index (BMI) are more susceptible to COVID-19. Public Health England (PHE) reported that having a BMI of 35 to 40 kg/m^2^ could increase the risk of death from COVID-19 by 40% and a BMI of greater than 40 by 90% (www.gov.uk/government/publications/excess-weight-and-covid-19-insights-from-new-evidence). Patients with obesity are often affected by respiratory dysfunction and disease, mainly related to inflammatory and immune function. The threat of severe COVID-19 to patients with obesity and impaired metabolic health (characterized by hypertension, dyslipidemia and hyperglycemia) is much higher than that of the general population [3,4]. Evidence from epidemiological observations and reporting data showed that 20%-50% of patients in the COVID-19 pandemic had diabetes [5], with the risk of a fatal outcome from COVID-19 up to 50% higher than those free of diabetes [6]. Low vitamin D status has also been examined whether it may be associated with increased susceptibility to COVID-19, as well as hospitalization and critical illness in patients with COVID-19 [7]. The proposed mechanisms are related to its anti-inflammatory properties, affecting lung capacity and lung function, but the reasons are still unclear [8,9]. Although studies have identified preexisting metabolic abnormalities, such as type 2 diabetes and hypertension, as the most common comorbidities for coronavirus infections [10,11], it remains unknown whether obesity, metabolic health and extended combinations of vitamin D sufficiency predispose individuals to develop COVID-19.

Here, we used the UK Biobank database to investigate whether metabolic/obesity phenotypes and vitamin D status have a role in COVID-19 hospitalization, detection, and severity. We pursued two hypotheses: 1) the health risk and illness severity for individuals with COVID-19 may increase disproportionately within different combinations of metabolic and obesity phenotypes; and 2) the discrepancy is likely to become even more pronounced when low vitamin D levels are present. Understanding how COVID-19 outcomes are associated with a broad combination of metabolic/obesity phenotypes and vitamin D status could inform future public health prevention programs, highlighting new at-risk individuals and populations.

## MATERIALS AND METHODS

### Study population

The UK Biobank is a prospective cohort study that recruited over half a million participants between 2006 and 2010 from across the UK [12]. The SARS-CoV-2 test information of UK Biobank participants from PHE has enabled researchers to investigate the risk factors for COVID-19 (www.bugbank.uk/index.html). We acquired the COVID-19 result data from March 16, 2020 to May 31, 2020. For the baseline enrollment, we excluded individuals whose locations were outside England, who died before the SARS-CoV-2 test, or who had missing data on the covariates included in the analysis.

### Exposure measures

Exposures were measured by the baseline information assessed during the initial enrollment. BMI was calculated as weight in kilograms divided by height in meters squared. Weight was measured to the nearest 0.1 kg using the Tanita BC-418 MA body composition analyzer. Height was measured using a Seca 202 height measure. We used metabolic disorders, hypertension, hypercholesterolemia, and diabetes to define metabolic health [13]. Hypertension was defined as a self-reported history of hypertension or systolic blood pressure greater than or equal to 140 mmHg or diastolic blood pressure greater than or equal to 90 mmHg or taking antihypertensive medications. Hypercholesterolemia was defined as a self-reported history of high cholesterol or taking medications. Diabetes was defined as a self-reported history of diabetes (type 1 or type 2 diabetes) or hospital records of diabetes or before recruitment (defined as ICD-10 codes E10-E14) or taking medications. According to the categories of BMI (normal weight [BMI 18.5-24.9 kg/m^2^], overweight [BMI 25.0-29.9 kg/m^2^], obesity [BMI ≥ 30.0 kg/m^2^]), and metabolic status (metabolically healthy [none of the metabolic disorders], metabolically unhealthy [at least one of the metabolic disorders]), participants were classified into six metabolic/obesity phenotypes, namely, metabolically healthy normal weight (MHNW), metabolically healthy overweight (MHOW), metabolically healthy obesity (MHO), metabolically unhealthy normal weight (MUHNW), metabolically unhealthy overweight (MUHOW), and metabolically unhealthy obesity (MUHO). Serum vitamin D concentration (nmol/L) was measured by CLIA analysis on a DiaSorin Ltd. We used two criteria to describe vitamin D status: vitamin D deficiency (< 25 nmol/L) and vitamin D insufficiency (< 50 nmol/L).

### Outcome measures

Investigated outcomes were COVID-19 hospitalization, detection, and severity. According to the COVID-19 data, including the specimen date, origin (the sample hospitalized with testing for SARS-CoV-2), and the result (positive or negative), we defined COVID-19 hospitalization as one record of origin (whether the patient was tested positive or not), confirmed COVID-19 as at least one positive test result. These hospitalized patients with SARS-CoV-2 positive infection were defined as severe COVID-19 [14].

### Covariates

Covariates included relevant demographic (age, sex, ethnicity), socio-economic (Townsend deprivation index, qualifications, employment), and behavioral (smoking status) factors. Age was calculated from birth to March 15, 2020 (a date before the first specimen test was reported). Ethnicity was self-reported and categorized as White, Black or Black British, Asian or Asian British, and mixed. Townsend deprivation index (TDI) was categorized into quintiles 1 to 5, and the higher quintile represents a more significant socio-economic deprivation. Qualifications were classified into seven categories: college degree, A-levels/AS-levels, O-levels/GCSEs, CSEs, NVQ/HND/HNC, other professional qualifications, and none of the above. The employment status was recoded as working, retired, unemployed, and others. Smoking status was self-reported and categorized as never, previous, and current smoker.

### Statistical analysis

The sample characteristics were presented as mean (standard deviation) or number (proportion). We used the Student's *t*-test for continuous variables, and chi-square test for categorical variables to compare differences between groups. Multivariable logistic regression analysis was performed to calculate odds ratios (ORs) and 95% confidence intervals (CIs), and assess the effects of metabolic/obesity phenotypes, vitamin D status and their combination on COVID-19 hospitalization, confirmed COVID-19 and COVID-19 severity. Logistic models were adjusted for sex, age, TDI, qualifications, employment, ethnicity, and smoking status. We conducted univariate logistic regression analysis and visually presented our subgroup analyses of the association between vitamin D insufficiency and reported COVID-19 outcomes. As sensitivity analyses, these models were repeated with participants stratified by sex (female or male) and age (< 65 or ≥ 65 years old). To assess the robustness of our findings, we examined the associations between MHO and COVID-19 using different definitions of metabolic health considering specific metabolic syndrome components (triglyceride, high-density lipoprotein cholesterol, blood pressure, and glucose levels), or further including waist circumference (WC) as a criterion [15,16] (Supplementary Text). All analyses were performed using Stata version 14.0, and all of the hypothesis testing was two-tailed with *P* < 0.05-set as nominal significance.

**Table 1:** Basic characteristics.

Characteristics	Total (N= 353,299)	COVID-19 hospitalization			Confirmed COVID-19			Severe COVID-19		
		No (n=349,797)	Yes (n=3,502)	*P-*value	No (n=352,217)	Yes (n=1,082)	*P-*value	No (n=352,585)	Yes (n=714)	*P-*value
Age (years)	67.7 (8.1)	67.7 (8.1)	69.3 (8.5)	<0.001	67.7 (8.1)	67 (9.3)	0.006	67.7 (8.1)	68.3 (9)	0.058
Age category				<0.001			0.957			<0.001
< 65	124,985 (35.4%)	123,941 (35.4%)	1,044 (29.8%)		124,524 (35.3%)	461 (42.6%)		124,734 (35.4%)	251 (35.1%)	
≥ 65	228,314 (64.6%)	225,856 (64.6%)	2,458 (70.2%)		227,693 (64.7%)	621 (57.4%)		227,851 (64.6%)	463 (64.9%)	
Female	192,001 (54.4%)	190,322 (54.4%)	1,679 (47.9%)	<0.001	191,475 (54.4%)	526 (48.6%)	<0.001	191,681 (54.4%)	320 (44.8%)	<0.001
Townsend deprivation index				<0.001			<0.001			<0.001
Q1 (lowest deprived)	70,881 (20.1%)	70,327 (20.1%)	554 (15.8%)		70,721 (20.1%)	160 (14.8%)		70,781 (20.1%)	100 (14%)	
Q2	70,800 (20%)	70,161 (20.1%)	639 (18.2%)		70,621 (20.1%)	179 (16.5%)		70,684 (20.1%)	116 (16.2%)	
Q3	70,307 (19.9%)	69,682 (19.9%)	625 (17.9%)		70,134 (19.9%)	173 (16%)		70,187 (19.9%)	120 (16.8%)	
Q4	70,762 (20%)	70,062 (20%)	700 (20%)		70,533 (20%)	229 (21.2%)		70,610 (20%)	152 (21.3%)	
Q5 (highest deprived)	70,549 (20%)	69,565 (19.9%)	984 (28.1%)		70,208 (19.9%)	341 (31.5%)		70,323 (19.9%)	226 (31.7%)	
Ethnicity				0.048			<0.001			<0.001
White	334,181 (94.6%)	330,901 (94.6%)	3,280 (93.7%)		333,235 (94.6%)	946 (87.4%)		333,556 (94.6%)	625 (87.5%)	
Black or black British	5,997 (1.7%)	5,919 (1.7%)	78 (2.2%)		5,940 (1.7%)	57 (5.3%)		5,965 (1.7%)	32 (4.5%)	
Asian or Asian British	7,688 (2.2%)	7,601 (2.2%)	87 (2.5%)		7,638 (2.2%)	50 (4.6%)		7,650 (2.2%)	38 (5.3%)	
Mixed	5,433 (1.5%)	5,376 (1.5%)	57 (1.6%)		5,404 (1.5%)	29 (2.7%)		5,414 (1.5%)	19 (2.7%)	
Employment				<0.001			0.025			0.003
Working	210,021 (59.4%)	208,316 (59.6%)	1,705 (48.7%)		209,410 (59.4%)	611 (56.5%)		209,642 (59.5%)	379 (53.1%)	
Retired	112,504 (31.8%)	111,114 (31.8%)	1,390 (39.7%)		112,154 (31.8%)	350 (32.4%)		112,251 (31.8%)	253 (35.4%)	
Unemployed	25,309 (7.2%)	24,957 (7.1%)	352 (10%)		25,209 (7.2%)	100 (9.2%)		25,241 (7.2%)	68 (9.5%)	
Other	5,465 (1.6%)	5,410 (1.5%)	55 (1.6%)		5,444 (1.6%)	21 (1.9%)		5,451 (1.5%)	14 (2%)	
Qualifications				<0.001			<0.001			<0.001
College degree	116,025 (32.8%)	115,100 (32.9%)	925 (26.4%)		115,759 (32.9%)	266 (24.6%)		115,857 (32.9%)	168 (23.5%)	
A levels/AS levels	40,289 (11.4%)	39,951 (11.4%)	338 (9.7%)		40,180 (11.4%)	109 (10.1%)		40,223 (11.4%)	66 (9.3%)	
O levels/GCESs	77,335 (21.9%)	76,619 (21.9%)	716 (20.4%)		77,132 (21.9%)	203 (18.7%)		77,200 (21.9%)	135 (18.9%)	
CSEs	21,001 (5.9%)	20,802 (6%)	199 (5.7%)		20,921 (5.9%)	80 (7.4%)		20,956 (5.9%)	45 (6.3%)	
NVQ or HND or HNC	23,500 (6.7%)	23,246 (6.6%)	254 (7.3%)		23,403 (6.6%)	97 (9%)		23,442 (6.6%)	58 (8.1%)	
Other professional qualifications	18,275 (5.2%)	18,081 (5.2%)	194 (5.5%)		18,212 (5.2%)	63 (5.8%)		18,232 (5.2%)	43 (6%)	
None of the above	56,874 (16.1%)	55,998 (16%)	876 (25%)		56,610 (16.1%)	264 (24.4%)		56,675 (16.1%)	199 (27.9%)	
Smoking status				<0.001			<0.001			<0.001
Never	196,688 (55.7%)	195,053 (55.8%)	1,635 (46.7%)		196,148 (55.7%)	540 (49.9%)		196,357 (55.7%)	331 (46.4%)	
Previous	122,615 (34.7%)	121,222 (34.6%)	1,393 (39.8%)		122,198 (34.7%)	417 (38.5%)		122,319 (34.7%)	296 (41.4%)	
Current	33,996 (9.6%)	33,522 (9.6%)	474 (13.5%)		33,871 (9.6%)	125 (11.6%)		33,909 (9.6%)	87 (12.2%)	
BMI (kg/m^2^)	27.4 (4.7)	27.4 (4.7)	28.6 (5.2)	<0.001	27.4 (4.7)	28.7 (5.3)	<0.001	27.4 (4.7)	28.8 (5.2)	<0.001
BMI category				<0.001			<0.001			<0.001
Normal weight (18.5-24.9)	116,757 (33%)	115,875 (33.1%)	882 (25.2%)		116,488 (33.1%)	269 (24.9%)		116,590 (33.1%)	167 (23.4%)	
Overweight (25.0-29.9)	151,555 (42.9%)	150,088 (42.9%)	1,467 (41.9%)		151,086 (42.9%)	469 (43.3%)		151,240 (42.9%)	315 (44.1%)	
Obese (≥ 30.0)	84,987 (24.1%)	83,834 (24%)	1,153 (32.9%)		84,643 (24%)	344 (31.8%)		84,755 (24%)	232 (32.5%)	
Hypertension	96,247 (27.2%)	94,883 (27.1%)	1,364 (39%)	<0.001	95,871 (27.2%)	376 (34.8%)	<0.001	95,979 (27.2%)	268 (37.5%)	<0.001
Hypercholesterolemia	64,375 (18.2%)	63,378 (18.1%)	997 (28.5%)	<0.001	64,114 (18.2%)	261 (24.1%)	<0.001	64,176 (18.2%)	199 (27.9%)	<0.001
Diabetes	16,585 (4.7%)	16,237 (4.6%)	348 (9.9%)	<0.001	16,490 (4.7%)	95 (8.8%)	<0.001	16,514 (4.7%)	71 (9.9%)	<0.001
Metabolic/obesity phenotypes				<0.001			<0.001			<0.001
MHNW	93,130 (26.4%)	92,486 (26.4%)	644 (18.4%)		92,935 (26.4%)	195 (18%)		93,015 (26.4%)	115 (16.1%)	
MHOW	98,460 (27.9%)	97,643 (27.9%)	817 (23.3%)		98,172 (27.9%)	288 (26.6%)		98,281 (27.9%)	179 (25.1%)	
MHO	41,480 (11.7%)	41,080 (11.8%)	400 (11.4%)		41,336 (11.7%)	144 (13.3%)		41,389 (11.7%)	91 (12.7%)	
MUHNW	23,627 (6.7%)	23,389 (6.7%)	238 (6.8%)		23,553 (6.7%)	74 (6.8%)		23,575 (6.7%)	52 (7.3%)	
MUHOW	53,095 (15%)	52,445 (15%)	650 (18.6%)		52,914 (15%)	181 (16.7%)		52,959 (15%)	136 (19%)	
MUHO	43,507 (12.3%)	42,754 (12.2%)	753 (21.5%)		43,307 (12.3%)	200 (18.5%)		43,366 (12.3%)	141 (19.8%)	
Vitamin D concentration (nmol/L)	49.6 (21)	49.6 (21)	47 (21.4)	<0.001	49.6 (21)	46.2 (21.4)	<0.001	49.6 (21)	46.3 (22.1)	<0.001
Vitamin D deficiency (< 25 nmol/L)	42,853 (12.1%)	42,284 (12.1%)	569 (16.3%)	<0.001	42,676 (12.1%)	177 (16.4%)	<0.001	42,726 (12.1%)	127 (17.8%)	<0.001
Vitamin D insufficiency (< 50 nmol/L)	188,888 (53.5%)	186,804 (53.4%)	2,084 (59.5%)	<0.001	188,221 (53.4%)	667 (61.7%)	<0.001	188,449 (53.5%)	439 (61.5%)	<0.001

Abbreviations: COVID-19, Coronavirus Disease 2019; BMI, body mass index; MHNW, metabolically healthy normal weight; MHO, metabolically healthy obesity; MHOW, metabolically healthy overweight; MUHNW, metabolically unhealthy normal weight; MUHO, metabolically unhealthy obesity; MUHOW, metabolically unhealthy overweight.

**Figure 1.**
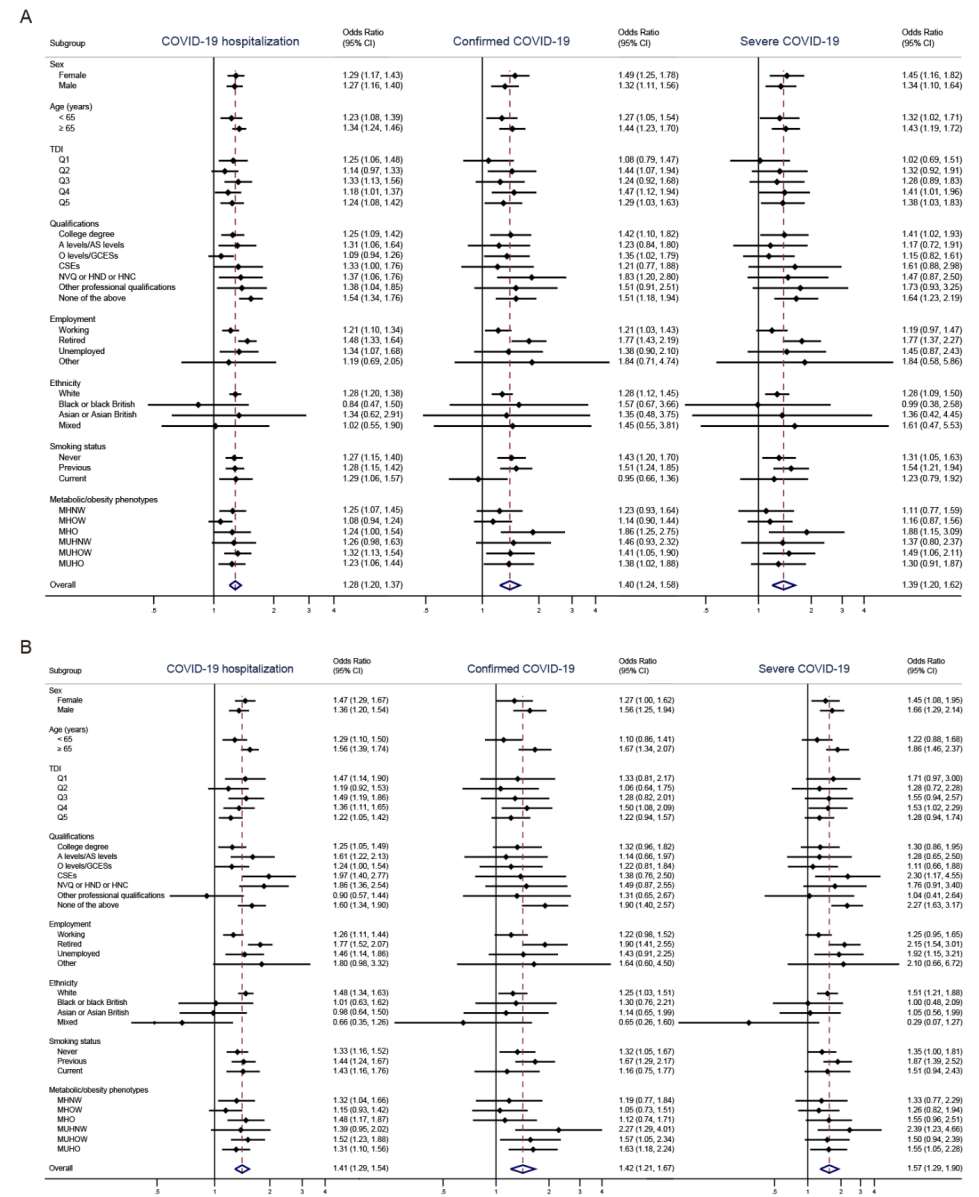
Association between (A) vitamin D insufficiency and (B) vitamin D deficiency (as exposure variable) and reported COVID-19 outcomes (as outcome) by subgroup analysis. The analysis used univariate logistic regression models. Abbreviations: COVID-19, Coronavirus Disease 2019; BMI, body mass index; CI, confidence interval; MHNW, metabolically healthy normal weight; MHO, metabolically healthy obesity; MHOW, metabolically healthy overweight; MUHNW, metabolically unhealthy normal weight; MUHO, metabolically unhealthy obesity; MUHOW, metabolically unhealthy overweight; TDI, Townsend deprivation index.

## RESULTS

### Baseline characteristics

The characteristics of participants by three COVID-19 outcomes are summarized in Table 1. The total sample included 353,299 adults with a mean age of 67.7±8.1 years. According to the SARS-CoV-2 test records, 3,502, 1,082, and 714 cases of COVID-19 hospitalization, confirmed COVID-19, and severe COVID-19 were determined, respectively. A total of 192,001 subjects (54.4%) were females; the percentage of women was lower than men in the three COVID-19 outcomes. A total of 228,314 subjects (64.6%) were ≥65 years old; the percentage of the elderly was different in the three COVID-19 outcomes being the highest in COVID-19 hospitalization and the lowest in confirmed COVID-19. The most deprived quintile had a higher prevalence of three COVID-19 outcomes than did the other quintiles. Almost two-thirds (67%) of adults in England are overweight or obese. Those of the black, and Asian ethnic groups are more severely affected. Overall, 96,247 (27.2%) participants were defined as hypertension category, 64,375 (18.2%) as hypercholesterolemia, 16,585 (4.7%) as diabetes, 41,480 (11.7%) as MHO, 43,507 (12.3%) as MUHO, and 188,888 (53.5%) as vitamin D insufficiency. Participants who were identified with COVID-19 hospitalization, confirmed COVID-19, and severe COVID-19 had higher percentages of obesity, smoking, MUHOW, and slightly lower vitamin D levels than negative ones.

**Table 2:** Comparison of multivariable ORs for obesity vs. normal-weight level of BMI for COVID-19, without and with adjustment for metabolic or vitamin D status.

Model adjusted^†^	COVID-19 hospitalization		Confirmed COVID-19		Severe COVID-19	
	OR (95% CI)	Difference in OR, %	OR (95% CI)	Difference in OR, %	OR (95% CI)	Difference in OR, %
Non metabolic & vitamin D status adjusted	1.56 (1.42, 1.70)	Ref.	1.48 (1.25, 1.74)	Ref.	1.53 (1.25, 1.88)	Ref.
Metabolic status adjusted	1.44 (1.31, 1.58)	7.7	1.39 (1.18, 1.65)	6.1	1.43 (1.16, 1.76)	6.5
Vitamin D status adjusted	1.50 (1.36, 1.64)	3.8	1.43 (1.22, 1.69)	3.4	1.49 (1.21, 1.83)	2.6
Both metabolic & vitamin D status adjusted	1.38 (1.26, 1.52)	11.5	1.35 (1.14, 1.60)	8.8	1.39 (1.12, 1.71)	9.2

† Basic model adjusted for sex, age, Townsend deprivation index, qualifications, employment, ethnicity, and smoking status. Abbreviations: COVID-19, Coronavirus Disease 2019; CI, confidence interval; OR, odds ratio.

### Metabolic/obesity phenotypes and COVID-19

Adjusting for metabolic health and vitamin D status attenuated the associations between obesity and COVID-19 outcomes compared to those of normal weight; however, participants with obesity were still consistent with a 38%, 35%, and 39% higher risk of COVID-19 hospitalization, confirmed COVID-19, and severe COVID-19, respectively (Table 2). Supplementary Table 1 presents the risk of three COVID-19 outcomes cross-classified by metabolic/obesity phenotypes. MHNW participants were used as the reference group. After adjusting for confounders, we observed a higher risk of all three COVID-19 outcomes among MHO participants (OR for COVID-19 hospitalization = 1.28, 95% CI, 1.13-1.46; OR for confirmed COVID-19 = 1.42, 95% CI, 1.14-1.76; OR for severe COVID-19 = 1.50, 95% CI, 1.14-1.98), as well as MUHO participants (OR for COVID-19 hospitalization = 1.96, 95% CI, 1.75-2.19; OR for confirmed COVID-19 = 1.83, 95% CI, 1.49-2.25; OR for severe COVID-19 = 1.94, 95% CI, 1.50-2.50).

### Vitamin D status and COVID-19

Figure 1A shows the associations between vitamin D insufficiency and reported COVID-19 outcomes by subgroup analysis. In the univariate models, vitamin D insufficiency was significantly associated with increased risk of COVID-19 hospitalization (OR = 1.28, 95% CI, 1.20-1.37), confirmed COVID-19 (OR = 1.40, 95% CI, 1.24-1.58), and severe COVID-19 (OR = 1.39, 95% CI, 1.20-1.62) overall, as well as within different sex- or age- levels. Similar relationships were found between vitamin D deficiency and COVID-19 (Fig. 1B). After adjustment for covariates, significant results covering all three COVID-19 outcomes remained for the category of vitamin D insufficiency (Supplementary Table 2).

### Metabolic/obesity phenotypes, vitamin D status, and COVID-19

**Figure 2.**
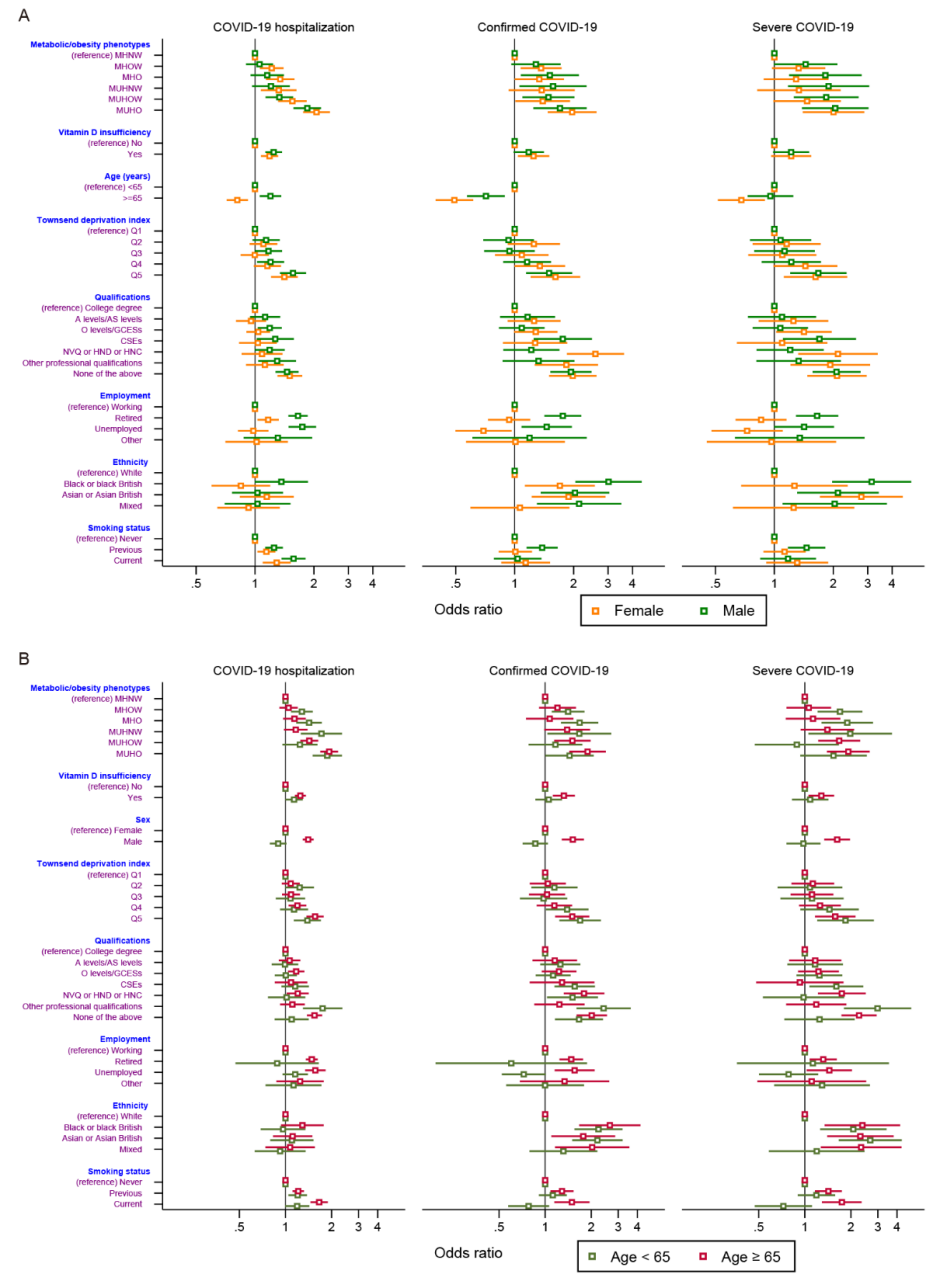
Associations of metabolic/obesity phenotypes and vitamin D status with COVID-19 outcomes stratified by (A) sex and (B) age subgroups. The models adjusted for age, Townsend deprivation index, qualifications, employment, ethnicity, and smoking status. Abbreviations: COVID-19, Coronavirus Disease 2019; MHNW, metabolically healthy normal weight; MHO, metabolically healthy obesity; MHOW, metabolically healthy overweight; MUHNW, metabolically unhealthy normal weight; MUHO, metabolically unhealthy obesity; MUHOW, metabolically unhealthy overweight.

Figure 2A presents sex differences in the associations of metabolic/obesity phenotypes and vitamin D levels with three COVID-19 outcomes. COVID-19 risk was especially high in the MUHO group for both men and women. We observed the harmful effects of vitamin D insufficiency with COVID-19 risk; however, the association was null for confirmed COVID-19 in men and severe COVID-19 in both men and women. For elderly female participants, protective effects were investigated against the three COVID-19 outcomes. Men showed higher risks of COVID-19 than women in the elderly sample (Fig. 2B). Similar trends were observed in the sensitivity analyses using the cutoff points of specific metabolic syndrome components and WC as definitions of metabolic health (Supplementary Fig. 1 and Fig. 2).

The ORs and CIs of the associations of metabolic/obesity phenotypes with and without vitamin D deﬁciency/insufﬁciency with three COVID-19 outcomes are shown in Table 3. The MUHO phenotype and vitamin D deﬁciency were associated with the highest risks of COVID-19 hospitalization (OR = 2.46, 95% CI, 2.05-2.94), confirmed COVID-19 (OR = 2.34, 95% CI, 1.69-3.23), and severe COVID-19 (OR = 2.48, 95% CI, 1.66-3.70). MUHO phenotype and vitamin D insufﬁciency were also associated with a high risk (OR for COVID-19 hospitalization = 2.33, 95% CI, 2.02-2.70; OR for confirmed COVID-19 = 2.06, 95% CI, 1.58-2.70; OR for severe COVID-19 = 2.06, 95% CI, 1.47-2.87). Furthermore, the results for sensitivity analyses with different definitions of metabolic status were consistent (Supplementary Table 3 and Supplementary Table 4).

**Table 3:** Joint associations of metabolic/obesity phenotypes and vitamin D status with COVID-19 outcomes.

Characteristics	Vitamin D deﬁciency (< 25 nmol/L)				Vitamin D insufﬁciency (< 50 nmol/L)			
	No		Yes		No		Yes	
	OR (95% CI)^†^	*P*	OR (95% CI)^†^	*P*	OR (95% CI)^†^	*P*	OR (95% CI)^†^	*P*
*COVID-19 hospitalization*								
MHNW	1 (Ref.)		1.24 (0.98, 1.57)	0.071	1 (Ref.)		1.24 (1.06, 1.45)	0.007
MHOW	1.14 (1.02, 1.27)	0.024	1.27 (1.03, 1.58)	0.027	1.20 (1.03, 1.40)	0.021	1.30 (1.12, 1.50)	<0.001
MHO	1.22 (1.06, 1.40)	0.006	1.77 (1.42, 2.22)	<0.001	1.23 (0.99, 1.52)	0.058	1.53 (1.30, 1.81)	<0.001
MUHNW	1.21 (1.02, 1.42)	0.024	1.52 (1.06, 2.18)	0.024	1.21 (0.98, 1.51)	0.079	1.49 (1.20, 1.84)	<0.001
MUHOW	1.36 (1.21, 1.54)	<0.001	1.97 (1.59, 2.46)	<0.001	1.33 (1.13, 1.58)	0.001	1.76 (1.50, 2.05)	<0.001
MUHO	1.91 (1.69, 2.15)	<0.001	2.46 (2.05, 2.94)	<0.001	1.88 (1.58, 2.24)	<0.001	2.33 (2.02, 2.70)	<0.001
*Confirmed COVID-19*								
MHNW	1 (Ref.)		0.93 (0.60, 1.44)	0.734	1 (Ref.)		1.11 (0.84, 1.47)	0.474
MHOW	1.31 (1.08, 1.59)	0.007	1.09 (0.75, 1.59)	0.657	1.34 (1.02, 1.75)	0.034	1.38 (1.07, 1.79)	0.015
MHO	1.42 (1.12, 1.80)	0.004	1.34 (0.89, 2.01)	0.167	1.02 (0.68, 1.52)	0.937	1.73 (1.31, 2.29)	<0.001
MUHNW	1.31 (0.97, 1.77)	0.078	2.21 (1.29, 3.78)	0.004	1.33 (0.89, 1.98)	0.168	1.71 (1.18, 2.48)	0.005
MUHOW	1.41 (1.12, 1.77)	0.003	1.72 (1.15, 2.57)	0.009	1.34 (0.98, 1.84)	0.065	1.70 (1.28, 2.26)	<0.001
MUHO	1.68 (1.34, 2.11)	<0.001	2.34 (1.69, 3.23)	<0.001	1.63 (1.16, 2.28)	0.004	2.06 (1.58, 2.70)	<0.001
*Severe COVID-19*								
MHNW	1 (Ref.)		1.05 (0.61, 1.81)	0.866	1 (Ref.)		1.00 (0.69, 1.45)	0.996
MHOW	1.32 (1.03, 1.70)	0.031	1.35 (0.86, 2.12)	0.196	1.27 (0.90, 1.79)	0.173	1.36 (0.98, 1.88)	0.067
MHO	1.42 (1.04, 1.93)	0.027	1.90 (1.19, 3.03)	0.007	0.99 (0.60, 1.65)	0.976	1.75 (1.24, 2.49)	0.002
MUHNW	1.38 (0.96, 2.00)	0.085	2.43 (1.29, 4.59)	0.006	1.37 (0.85, 2.22)	0.195	1.65 (1.04, 2.60)	0.033
MUHOW	1.58 (1.19, 2.09)	0.001	1.86 (1.15, 3.02)	0.012	1.35 (0.92, 1.97)	0.125	1.82 (1.29, 2.57)	0.001
MUHO	1.82 (1.37, 2.41)	<0.001	2.48 (1.66, 3.70)	<0.001	1.68 (1.12, 2.52)	0.012	2.06 (1.47, 2.87)	<0.001

† Adjusted for sex, age, Townsend deprivation index, qualifications, employment, ethnicity, and smoking status. Abbreviations: COVID-19, Coronavirus Disease 2019; CI, confidence interval; MHNW, metabolically healthy normal weight; MHO, metabolically healthy obesity; MHOW, metabolically healthy overweight; MUHNW, metabolically unhealthy normal weight; MUHO, metabolically unhealthy obesity; MUHOW, metabolically unhealthy overweight; OR, odds ratio.

## DISCUSSION

In this study, we conducted a combined analysis of metabolic/obesity phenotypes and vitamin D status related to COVID-19 using a total sample of 353,299 adults in England. Our study found that being the MUHO phenotype and low vitamin D status could highly increase the risk of detection and severe illness from COVID-19. In addition, men in the elderly participants tended to have higher susceptibility and severity for the three COVID-19 categories (COVID-19 hospitalization, confirmed COVID-19, or severe COVID-19).

The association between excess body weight and metabolic disorders has been confirmed [17]. In our study, nearly a quarter of the total subjects were affected by obesity, among which over half were defined as MUHO. Our study took MUHO as an aggregative indicator to expand the obesity pattern by adding its metabolic phenotypes. The MHO phenotype had the highest risks of COVID-19 hospitalization, confirmed COVID-19, and severe COVID-19, and these risks were also observed for the MUHO phenotype. These findings agree with studies showing the metabolic and endocrine mechanistic link to coronavirus infections [18]. Initial reports and systematic reviews have documented the close connections of fat distribution, diabetes, and metabolic syndrome with critical illness and prognosis in COVID-19 [19-21]. With the substantial increases in prevalence worldwide, obesity and related metabolic abnormalities have become major concerns for chronic non-communicable diseases and infectious diseases [22]. COVID-19 outcomes are critically related to patients' health conditions, and thus more attention should be given to these high-risk groups, which brings important public health challenges.

The multiple effects of vitamin D on respiratory illnesses or other diseases and conditions have been investigated. A systematic review and meta-analysis, including 11,321 participants, examined the protected impact of vitamin D supplementation on acute respiratory tract infections, namely, pneumonia, bronchitis, and sinusitis [23]. As a respiratory disease caused by the novel coronavirus, COVID-19 may be harder to be overcome for people with vitamin D deficiency or insufficiency. Preliminary research has examined the possible protective role of vitamin D concentration for COVID-19 using the UK Biobank data. However, no significant association between vitamin D and COVID-19 was found after adjusting for potential confounders [24].

Our study has updated the data, set more restrictions on sample selection at baseline, and expanded the COVID-19 outcomes. The number of confirmed cases of COVID-19 included in this previous study was 449 cases; to better explain the difference, we reset our cutoff testing date, got a similar case number, repeated our analysis, and obtained identical nonsignificant results (Supplementary Table 5). However, in the current study with 1,082 confirmed cases, our findings showed a significantly positive association between vitamin D insufficiency and COVID-19 hospitalization, confirmed COVID-19, and severe COVID-19 in the elderly patients adjusted by the metabolic/obesity phenotypes and other covariates or not. As small sample sizes may lead to bias, we inferred that the increased case numbers might alter the observed associations and improve accuracy.

Moreover, our study indicated that vitamin D status might moderate the associations between metabolic/ obesity phenotypes and COVID-19 outcomes. There are several pathways by which metabolic/obesity phenotypes may be related to COVID-19; obesity and metabolic disorders may directly contribute to COVID-19 severity, or they may be involved in other causal relationships. A large number of observational studies have demonstrated the role of vitamin D status in metabolic processes and obesity [25], suggesting that lower levels of vitamin D might be implicated in possible mechanisms underlying increased risk of COVID-19 severity in metabolism impairment.

Even though our work demonstrated that obesity accounted for a larger proportion of positive infections from SARS-CoV-2, it does not suggest that having excess weight can increase the chances of contracting COVID-19. However, the current evidence does show that patients with these metabolic/obesity phenotypes and low vitamin D status are significantly more likely to be admitted to intensive care with COVID-19 and become seriously ill compared to those with a healthier situation. Because of the global spread of pandemic COVID-19, the World Health Organization (WHO) has warned that most people are still susceptible. As a new infectious disease, COVID-19 can affect anyone, and the disease can cause symptoms ranging from mild to severe. Thus, more work is needed to better understand the potential risk factors for severe illness or complications. To fight this new virus and reduce losses due to disease, there is a great need for more intensive detection and sustainable behavioral interventions focused on high-risk people.

Sex differences were observed in our study, and it seemed that women had better outcomes than men in the detection or severity of COVID-19, especially in the older sample subjects (≥ 65 years old). Consistent with previous reports, men infected with COVID-19 were more likely to develop more serious cases and a higher mortality than women [26-28]. An observational cohort study collecting the clinical features of 20,133 UK patients in the hospital with COVID-19 found 20% more men admitted to hospitals than women [29]. In addition, the sex and gender disparities were also observed in the severe acute respiratory syndrome coronavirus (SARS-CoV) and the Middle East respiratory syndrome coronavirus; these two similar coronaviruses were found to infect more men than women both *in vivo* and *in vitro* studies [30-32]. Both congenital and acquired conditions might explain that men seemed more prone to these infections than women. Previous studies have demonstrated that men had a relatively higher expression of angiotensin-converting enzyme 2 (ACE2), an enzymatic system also modulated by sex hormones, which has been established as the functional host receptor for SARS-CoV-2 [33,34]. As for physical, behavioral, and social factors, men were found to have a higher contribution of preexisting diseases, higher smoking rates, higher likelihood to work outside the home, and lower likelihood to follow hand hygiene practices and seek preventive care than women [35,36]. To better understand the gender differences and devise a personalized health monitoring system, it is important to acquire more evidence from established population-based studies and laboratory tests.

To our knowledge, relationships between obesity, metabolic health and extended combinations of vitamin D insufficiency have not been investigated. The results highlight that supporting people to maintain a healthy weight, protected against metabolic diseases, and achieving adequate vitamin D levels may reduce the severe effects of COVID-19, especially among the elderly population. Our study has several limitations. First, a retrospective study's inherent limitation makes it impossible to infer causality in the association between metabolic/obesity phenotypes, vitamin D status, and the risk of COVID-19. Second, even if we use multiple statistical models to adjust for potential confounders, some unmeasured and unforeseen confounding factors may still potentially affect the magnitude of metabolic and vitamin D effects on COVID-19 outcomes. Third, BMI, metabolic or vitamin D status may change over time in a substantial proportion of the population; however, our study did not reflect longitudinal changes in measurements and illustrated the underlying assumption and relevance. Moreover, we chose relatively fixed covariates that do not easily change with time to ensure statistical models’ stability.

In conclusion, metabolic/obesity phenotypes and vitamin D status are differentially associated with the development of COVID-19 in adults. In addition, obesity with a combination of metabolic disorders and vitamin D insufficiency could highly increase the risk of detection and severe illness from COVID-19. Such indicators might be useful in a primary care setting and in a hospital setting to assess the risk of a complicated course of disease in patients with a positive SARS-CoV-2 test. Additional research will help us confirm if these are risk factors for severe COVID-19 illness and determine whether other factors increase a person's risk.

## Supplementary Materials

The Supplemenantry data can be found online at: www.aginganddisease.org/EN/10.14336/AD.2020.1108.


