# Supplementary material for: Metabolic Healthy Obesity, Vitamin D Status, and Risk of COVID-19
Source: Aging Dis. 2021 Feb 1;12(1):61–71. doi: 10.14336/AD.2020.1108 (PMC7801267; doi:10.14336/AD.2020.1108)
Supplement: Supplementary file 1 [file AD-12-1-61-S.pdf]

Metabolic Healthy Obesity, Vitamin D Status, and Risk of COVID-19

Shu Li[1], Zhi Cao[1], Hongxi Yang[1,2], Yuan Zhang[1], Fusheng Xu[1], Yaogang Wang[1*]

© 2020. Li S et al. Published online at http://www.aginganddisease.org/EN/10.14336/AD.2020.1108

Supplementary Text.

Definitions of metabolic health using the cutoff points of specific metabolic syndrome components and waist circumference.

1. Under our definition of metabolic health using the cutoff points of specific metabolic syndrome components, participants having less than two of the four criteria were considered to be metabolic health[1], as follows: (1) normal triglyceride concentrations, defined as triglycerides <1.7 mmol/L (<150 mg/dL) and not on drug treatment for elevated triglyceride concentration at baseline; (2) normal high-density lipoprotein cholesterol (HDL-c) concentrations, defined as ⩾1.05 mmol/L (40 mg/dL) for men and ⩾1.25 mmol/L (50 mg/dL) for women; (3) normal blood pressure (BP), defined as a systolic BP <130 and a diastolic BP <85 mmHg, and not on antihypertensive drug treatment at baseline; (4) normal fasting glucose, defined as fasting blood glucose <5.6 mmol/L (100 mg/dL) and not on drug treatment at baseline.

2. Under our definition of metabolic health using the cutoff points of specific metabolic syndrome components plus waist circumference, participants having less than three of the five criteria were considered to be metabolic health, as follows: (1) normal triglyceride concentrations, defined as triglycerides <1.7 mmol/L (<150 mg/dL) and not on drug treatment for elevated triglyceride concentration at baseline; (2) normal high-density lipoprotein cholesterol (HDL-c) concentrations, defined as ⩾1.05 mmol/L (40 mg/dL) for men and ⩾1.25 mmol/L (50 mg/dL) for women; (3) normal blood pressure (BP), defined as a systolic BP <130 and a diastolic BP <85 mmHg, and not on antihypertensive drug treatment at baseline; (4) normal fasting glucose, defined as fasting blood glucose <5.6 mmol/L (100 mg/dL) and not on drug treatment at baseline; (5) waist circumference (WC), defined as WC <102 cm in men and <88 cm in women.

Reference

[1] Stefan N, Häring HU, Schulze MB. Metabolically healthy obesity: the low-hanging fruit in obesity treatment? Lancet Diabetes Endocrinol. 2018;6(3):249-258.

© 2020. Li S et al. Published online at http://www.aginganddisease.org/EN/10.14336/AD.2020.1108

Supplementary Table 1. The associations of metabolic/obesity phenotypes with COVID-19 outcomes.

Characteristics	Model 1†		Model 2‡	
	OR (95% CI)	*P*-value	OR (95% CI)	*P*-value
**COVID-19 hospitalization**				
**Metabolic/obesity phenotypes**				
MHNW	1 (Ref.)		1 (Ref.)	
MHOW	1.15 (1.03, 1.27)	0.010	1.13 (1.01, 1.25)	0.026
MHO	1.37 (1.21, 1.56)	<0.001	1.28 (1.13, 1.46)	<0.001
MUHNW	1.29 (1.11, 1.50)	0.001	1.21 (1.04, 1.41)	0.013
MUHOW	1.52 (1.36, 1.71)	<0.001	1.40 (1.25, 1.57)	<0.001
MUHO	2.24 (2.01, 2.50)	<0.001	1.96 (1.75, 2.19)	<0.001
**Confirmed COVID-19**				
**Metabolic/obesity phenotypes**				
MHNW	1 (Ref.)		1 (Ref.)	
MHOW	1.37 (1.14, 1.65)	0.001	1.29 (1.08, 1.56)	0.006
MHO	1.63 (1.31, 2.02)	<0.001	1.42 (1.14, 1.76)	0.002
MUHNW	1.63 (1.24, 2.14)	<0.001	1.44 (1.10, 1.90)	0.009
MUHOW	1.73 (1.41, 2.14)	<0.001	1.47 (1.18, 1.81)	<0.001
MUHO	2.32 (1.89, 2.84)	<0.001	1.83 (1.49, 2.25)	<0.001
**Severe COVID-19**				
**Metabolic/obesity phenotypes**				
MHNW	1 (Ref.)		1 (Ref.)	
MHOW	1.39 (1.10, 1.77)	0.006	1.32 (1.04, 1.67)	0.022
MHO	1.72 (1.31, 2.27)	<0.001	1.50 (1.14, 1.98)	0.004
MUHNW	1.73 (1.24, 2.41)	0.001	1.51 (1.08, 2.11)	0.015
MUHOW	1.92 (1.48, 2.48)	<0.001	1.61 (1.24, 2.09)	<0.001
MUHO	2.48 (1.92, 3.19)	<0.001	1.94 (1.50, 2.50)	<0.001

†Adjusted for sex, age. ‡Adjusted for sex, age, Townsend deprivation index, qualifications, employment, ethnicity, and smoking status. Abbreviations: COVID-19, Coronavirus Disease 2019; CI, confidence interval; MHNW, metabolically healthy normal weight; MHO, metabolically healthy obesity; MHOW, metabolically healthy overweight; MUHNW, metabolically unhealthy normal weight; MUHO, metabolically unhealthy obesity; MUHOW, metabolically unhealthy overweight; OR, odds ratio.

© 2020. Li S et al. Published online at http://www.aginganddisease.org/EN/10.14336/AD.2020.1108

Supplementary Table 2. The associations of vitamin D status with COVID-19 outcomes.

**Characteristics**	**Model 1**†		**Model 2**‡	
	**OR (95% CI)**	***P*-value**	**OR (95% CI)**	***P*-value**
	**COVID-19 hospitalization**			
**Vitamin D status**				
Vitamin D concentration (nmol/L)	0.993 (0.991, 0.994)	<0.001	0.996 (0.994, 0.997)	<0.001
Vitamin D deficiency (< 25 nmol/L)	1.500 (1.370, 1.643)	<0.001	1.292 (1.175, 1.420)	<0.001
Vitamin D insufficiency (< 50 nmol/L)	1.337 (1.249, 1.431)	<0.001	1.214 (1.132, 1.303)	<0.001
	**Confirmed COVID-19**			
**Vitamin D status**				
Vitamin D concentration (nmol/L)	0.992 (0.990, 0.995)	<0.001	0.997 (0.994, 1.000)	0.039
Vitamin D deficiency (< 25 nmol/L)	1.388 (1.180, 1.632)	<0.001	1.090 (0.919, 1.294)	0.321
Vitamin D insufficiency (< 50 nmol/L)	1.381 (1.221, 1.562)	<0.001	1.204 (1.059, 1.368)	0.005
	**Severe COVID-19**			
**Vitamin D status**				
Vitamin D concentration (nmol/L)	0.992 (0.988, 0.995)	<0.001	0.997 (0.993, 1.000)	0.088
Vitamin D deficiency (< 25 nmol/L)	1.604 (1.322, 1.946)	<0.001	1.227 (1.000, 1.505)	0.050
Vitamin D insufficiency (< 50 nmol/L)	1.413 (1.214, 1.644)	<0.001	1.206 (1.030, 1.412)	0.020

†Adjusted for sex, age. ‡Adjusted for sex, age, Townsend deprivation index, qualifications, employment, ethnicity, smoking status, and metabolic/obesity phenotypes. Abbreviations: COVID-19, Coronavirus Disease 2019; CI, confidence interval; OR, odds ratio.

Supplementary Table 3. Joint associations of metabolic/obesity phenotypes and vitamin D status with COVID-19 outcomes using the cutoff points of specific metabolic syndrome components as the definition of metabolic health.

Characteristics	Vitamin D deficiency (< 25 nmol/L)				Vitamin D insufficiency (< 50 nmol/L)			
	No		Yes		No		Yes	
	OR (95% CI)†	*P*-value	OR (95% CI)†	*P*-value	OR (95% CI)†	*P*-value	OR (95% CI)†	*P*-value
COVID-19 hospitalization								
MHNW	1 (Ref.)		1.25 (0.87, 1.79)	0.233	1 (Ref.)		1.28 (1.00, 1.63)	0.046
MHOW	1.30 (1.08, 1.56)	0.005	1.50 (1.04, 2.14)	0.028	1.30 (1.01, 1.67)	0.041	1.60 (1.26, 2.03)	<0.001
MHO	1.22 (0.95, 1.57)	0.122	1.91 (1.27, 2.85)	0.002	1.10 (0.74, 1.64)	0.648	1.68 (1.27, 2.23)	<0.001
MUHNW	0.93 (0.79, 1.09)	0.383	1.11 (0.85, 1.46)	0.438	0.96 (0.77, 1.19)	0.688	1.14 (0.92, 1.42)	0.215
MUHOW	1.05 (0.91, 1.22)	0.507	1.34 (1.09, 1.66)	0.006	1.10 (0.90, 1.34)	0.348	1.27 (1.05, 1.55)	0.015
MUHO	1.44 (1.24, 1.68)	<0.001	1.98 (1.64, 2.39)	<0.001	1.45 (1.17, 1.79)	0.001	1.82 (1.50, 2.21)	<0.001
Confirmed COVID-19								
MHNW	1 (Ref.)		0.79 (0.39, 1.57)	0.495	1 (Ref.)		0.98 (0.64, 1.49)	0.914
MHOW	1.49 (1.10, 2.01)	0.009	1.25 (0.69, 2.26)	0.465	1.39 (0.92, 2.11)	0.114	1.55 (1.05, 2.28)	0.028
MHO	1.31 (0.87, 1.96)	0.202	0.76 (0.30, 1.88)	0.545	0.88 (0.43, 1.80)	0.725	1.38 (0.86, 2.21)	0.183
MUHNW	0.95 (0.71, 1.26)	0.704	1.19 (0.75, 1.88)	0.458	0.89 (0.61, 1.30)	0.550	1.09 (0.75, 1.56)	0.660
MUHOW	1.08 (0.84, 1.40)	0.545	1.10 (0.75, 1.60)	0.630	1.04 (0.74, 1.46)	0.826	1.14 (0.82, 1.59)	0.436

MUHO	1.33 (1.02, 1.74)	0.035	1.80 (1.30, 2.51)	<0.001	1.11 (0.76, 1.62)	0.580	1.60 (1.15, 2.22)	0.005
Severe COVID-19								
MHNW	1 (Ref.)		0.60 (0.22, 1.68)	0.335	1 (Ref.)		0.88 (0.50, 1.54)	0.660
MHOW	1.37 (0.92, 2.04)	0.121	1.64 (0.82, 3.27)	0.164	1.17 (0.68, 2.04)	0.571	1.58 (0.96, 2.60)	0.070
MHO	1.20 (0.69, 2.08)	0.513	0.79 (0.25, 2.57)	0.701	0.47 (0.14, 1.57)	0.221	1.43 (0.78, 2.61)	0.244
MUHNW	0.90 (0.63, 1.30)	0.584	1.29 (0.74, 2.24)	0.372	0.89 (0.55, 1.42)	0.614	1.00 (0.63, 1.59)	0.993
MUHOW	1.08 (0.78, 1.51)	0.631	1.15 (0.72, 1.83)	0.564	1.01 (0.65, 1.56)	0.961	1.14 (0.75, 1.73)	0.551
MUHO	1.33 (0.94, 1.87)	0.105	2.03 (1.35, 3.04)	0.001	1.16 (0.73, 1.85)	0.537	1.59 (1.04, 2.41)	0.031

†Adjusted for sex, age, Townsend deprivation index, qualifications, employment, ethnicity, and smoking status. Abbreviations: COVID-19, Coronavirus Disease 2019; CI, confidence interval; MHNW, metabolically healthy normal weight; MHO, metabolically healthy obesity; MHOW, metabolically healthy overweight; MUHNW, metabolically unhealthy normal weight; MUHO, metabolically unhealthy obesity; MUHOW, metabolically unhealthy overweight; OR, odds ratio.

Supplementary Table 4. Joint associations of metabolic/obesity phenotypes and vitamin D status with COVID-19 outcomes using the cutoff points of specific metabolic syndrome components plus waist circumference as the definition of metabolic health.

**Characteristics**	**Vitamin D deficiency (< 25 nmol/L)**				**Vitamin D insufficiency (< 50 nmol/L)**			
	**No**		**Yes**		**No**		**Yes**	
	**OR (95% CI)†**	***P*-value**	**OR (95% CI)†**	***P*-value**	**OR (95% CI)†**	***P*-value**	**OR (95% CI)†**	***P*-value**
**COVID-19 hospitalization**								
MHNW	1 (Ref.)		1.23 (0.97, 1.57)	0.084	1 (Ref.)		1.23 (1.05, 1.44)	0.010
MHOW	1.19 (1.06, 1.34)	0.004	1.34 (1.05, 1.70)	0.017	1.18 (1.01, 1.39)	0.042	1.42 (1.22, 1.65)	<0.001
MHO	1.26 (1.03, 1.53)	0.025	1.83 (1.28, 2.62)	0.001	1.32 (0.99, 1.77)	0.060	1.54 (1.22, 1.93)	<0.001
MUHNW	1.00 (0.84, 1.19)	0.997	1.15 (0.77, 1.71)	0.494	0.98 (0.78, 1.25)	0.887	1.22 (0.97, 1.52)	0.087
MUHOW	1.12 (0.99, 1.27)	0.061	1.52 (1.23, 1.88)	<0.001	1.15 (0.98, 1.36)	0.089	1.35 (1.16, 1.57)	<0.001
MUHO	1.56 (1.39, 1.75)	<0.001	2.10 (1.79, 2.47)	<0.001	1.52 (1.28, 1.79)	<0.001	1.92 (1.68, 2.21)	<0.001
**Confirmed COVID-19**								
MHNW	1 (Ref.)		0.93 (0.60, 1.44)	0.740	1 (Ref.)		1.01 (0.76, 1.34)	0.942
MHOW	1.24 (1.01, 1.53)	0.038	0.89 (0.57, 1.40)	0.622	1.15 (0.87, 1.53)	0.327	1.27 (0.97, 1.66)	0.082
MHO	1.37 (0.99, 1.90)	0.058	0.83 (0.39, 1.78)	0.636	1.16 (0.69, 1.94)	0.577	1.36 (0.93, 1.99)	0.117
MUHNW	1.02 (0.73, 1.41)	0.918	1.62 (0.90, 2.93)	0.110	0.84 (0.53, 1.34)	0.467	1.37 (0.93, 2.01)	0.107
MUHOW	1.20 (0.97, 1.49)	0.101	1.53 (1.06, 2.21)	0.023	1.12 (0.83, 1.51)	0.466	1.36 (1.04, 1.78)	0.023
MUHO	1.44 (1.17, 1.77)	0.001	1.88 (1.41, 2.52)	<0.001	1.18 (0.86, 1.61)	0.307	1.72 (1.34, 2.19)	<0.001
**Severe COVID-19**								
MHNW	1 (Ref.)		0.91 (0.52, 1.59)	0.738	1 (Ref.)		0.90 (0.63, 1.29)	0.569
MHOW	1.20 (0.93, 1.56)	0.162	1.10 (0.65, 1.85)	0.727	1.02 (0.72, 1.45)	0.922	1.26 (0.91, 1.75)	0.172
MHO	1.27 (0.83,	0.267	0.81 (0.30,	0.674	0.82 (0.39,	0.598	1.32 (0.82,	0.255

	1.94)		2.20)		1.71)		2.14)	
MUHNW	0.82 (0.54, 1.26)	0.363	1.71 (0.86, 3.39)	0.129	0.67 (0.37, 1.21)	0.182	1.13 (0.70, 1.83)	0.609
MUHOW	1.14 (0.87, 1.49)	0.329	1.51 (0.97, 2.35)	0.071	1.02 (0.71, 1.47)	0.903	1.24 (0.89, 1.72)	0.199
MUHO	1.39 (1.08, 1.80)	0.012	2.07 (1.47, 2.93)	<0.001	1.16 (0.80, 1.69)	0.424	1.61 (1.19, 2.17)	0.002

[†]Adjusted for sex, age, Townsend deprivation index, qualifications, employment, ethnicity, and smoking status. Abbreviations: COVID-19, Coronavirus Disease 2019; CI, confidence interval; MHNW, metabolically healthy normal weight; MHO, metabolically healthy obesity; MHOW, metabolically healthy overweight; MUHNW, metabolically unhealthy normal weight; MUHO, metabolically unhealthy obesity; MUHOW, metabolically unhealthy overweight; OR, odds ratio.

Supplementary Table 5. The comparison of results regarding the difference between our data and the published paper in the role of vitamin D status in COVID-19.

	**Hastie *et al.*[†]**		**Our data redefined [‡]**	
	**OR (95% CI)**	***P*-value**	**OR (95% CI)**	***P*-value**
Vitamin D concentration (nmol/L)	1.00 (0.998, 1.01)	0.208	0.997 (0.992, 1.002)	0.240
Vitamin D deficiency (< 25 nmol/L)	0.92 (0.71, 1.21)	0.564	1.080 (0.830, 1.407)	0.566
Vitamin D insufficiency (< 50 nmol/L)	0.88 (0.72, 1.08)	0.232	1.136 (0.927, 1.392)	0.219

[†]Adjusted for ethnicity, sex, month of assessment, Townsend deprivation quintile, household income, self-reported health rating, smoking status, BMI category, age at assessment, diabetes, SBP, DBP, and long-standing illness, disability or infirmity. Acquired from the Table 2 of the previous paper published at Diabetes Metab Syndr. 2020 July-August; 14(4): 561–565. [‡]Adjusted for sex, age, Townsend deprivation index, qualifications, employment, ethnicity, smoking status, and metabolic/obesity phenotypes. Our data redefined a total of 1481 individuals (versus 1474 individuals in Hastie *et al.*) with available SARS-CoV-2 test results by resetting the cutoff testing date.

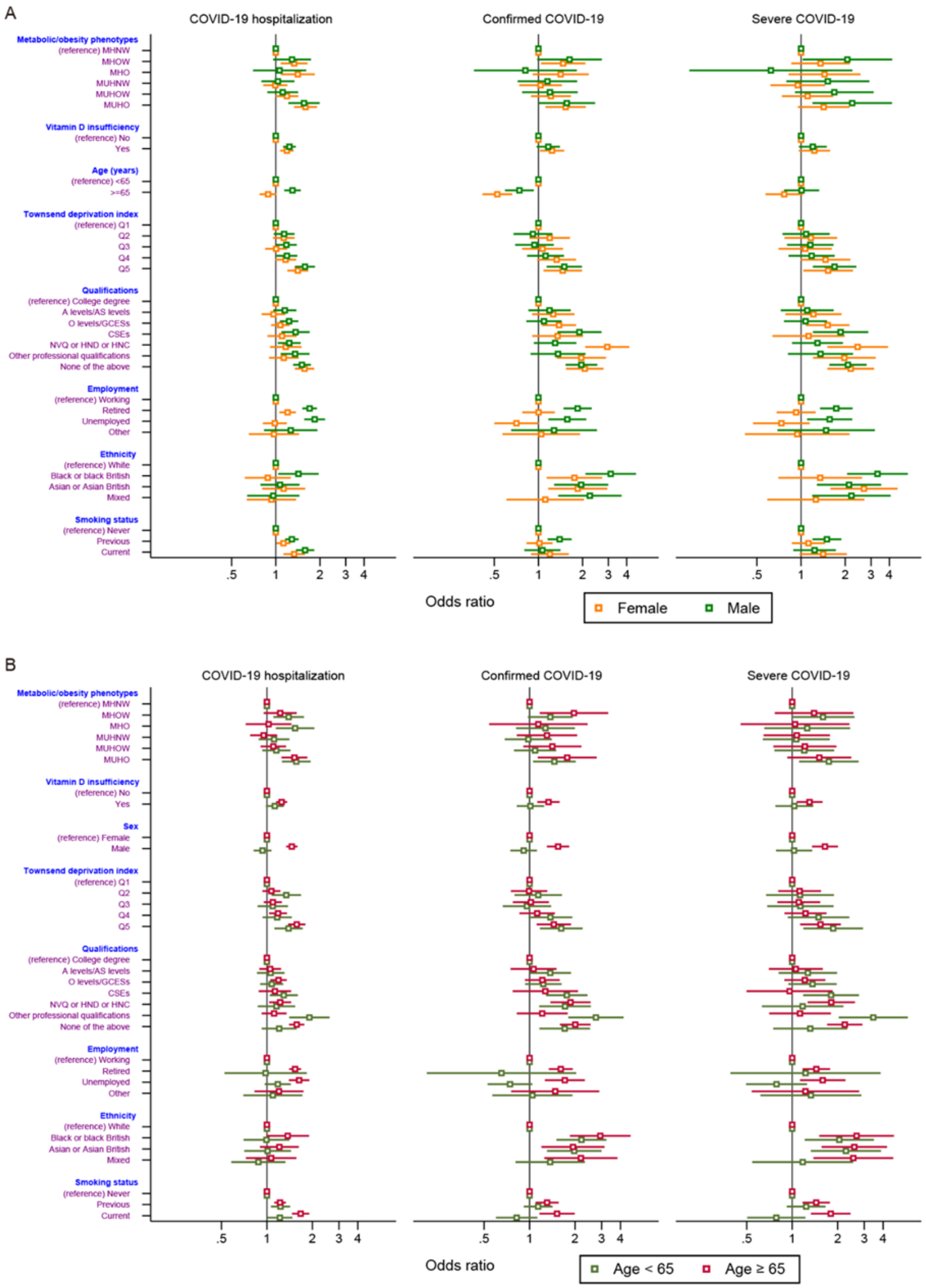

Supplementary Figure 1. Associations of metabolic/obesity phenotypes and vitamin D status with COVID-19 outcomes using the cutoff points of specific metabolic syndrome components as the definition of metabolic health stratified by (A) sex and (B) age subgroups. The models adjusted for age, Townsend deprivation index, qualifications, employment, ethnicity, and smoking status. Abbreviations: COVID-19, Coronavirus Disease 2019; MHNW, metabolically healthy normal weight; MHO, metabolically healthy obesity; MHOW, metabolically healthy overweight; MUHNW, metabolically unhealthy normal weight; MUHO, metabolically unhealthy obesity; MUHOW, metabolically unhealthy overweight.

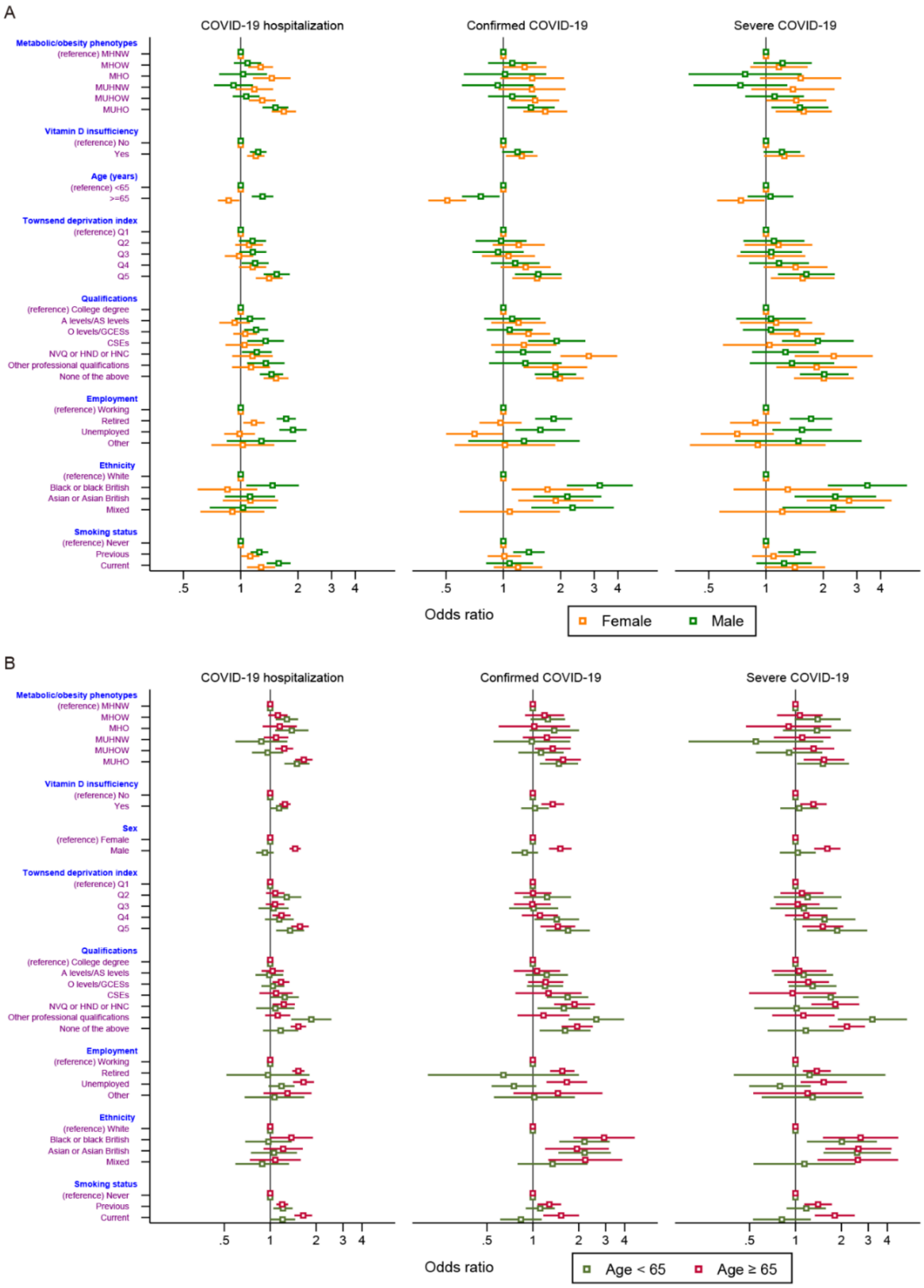

Supplementary Figure 2. Associations of metabolic/obesity phenotypes and vitamin D status with COVID-19 outcomes using the cutoff points of specific metabolic syndrome components plus waist circumference as the definition of metabolic health stratified by (A) sex and (B) age subgroups. The models adjusted for age, Townsend deprivation index, qualifications, employment, ethnicity, and smoking status. Abbreviations: COVID-19, Coronavirus Disease 2019; MHNW, metabolically healthy normal weight; MHO, metabolically healthy obesity; MHOW, metabolically healthy overweight; MUHNW, metabolically unhealthy normal weight; MUHO, metabolically unhealthy obesity; MUHOW, metabolically unhealthy overweight.
